# Development of New Modular Genetic Tools for Engineering the Halophilic Archaeon *Halobacterium salinarum*

**DOI:** 10.1371/journal.pone.0129215

**Published:** 2015-06-10

**Authors:** Rafael Silva-Rocha, Marjorie Cornejo Pontelli, Gilvan Pessoa Furtado, Livia Soares Zaramela, Tie Koide

**Affiliations:** Department of Biochemistry and Immunology–Ribeirão Preto Medical School, University of São Paulo, Ribeirão Preto, SP, Brazil; The Roslin Institute, UNITED KINGDOM

## Abstract

Our ability to genetically manipulate living organisms is usually constrained by the efficiency of the genetic tools available for the system of interest. In this report, we present the design, construction and characterization of a set of four new modular vectors, the pHsal series, for engineering *Halobacterium salinarum*, a model halophilic archaeon widely used in systems biology studies. The pHsal shuttle vectors are organized in four modules: (i) the *E*. *coli*’s specific part, containing a ColE1 origin of replication and an ampicillin resistance marker, (ii) the resistance marker and (iii) the replication origin, which are specific to *H*. *salinarum* and (iv) the cargo, which will carry a sequence of interest cloned in a multiple cloning site, flanked by universal M13 primers. Each module was constructed using only minimal functional elements that were sequence edited to eliminate redundant restriction sites useful for cloning. This optimization process allowed the construction of vectors with reduced sizes compared to currently available platforms and expanded multiple cloning sites. Additionally, the strong constitutive promoter of the *fer2* gene was sequence optimized and incorporated into the platform to allow high-level expression of heterologous genes in *H*. *salinarum*. The system also includes a new minimal suicide vector for the generation of knockouts and/or the incorporation of chromosomal tags, as well as a vector for promoter probing using a GFP gene as reporter. This new set of optimized vectors should strongly facilitate the engineering of *H*. *salinarum* and similar strategies could be implemented for other archaea.

## Introduction

Archaea have been recognized as an evolutionary independent domain of life only a few decades ago, presenting unique characteristics not existing in Eukarya or Bacteria, such as the presence of ether lipids in the membrane [1]. These organisms are well known for thriving in extreme environments such as high temperatures, low pHs, high salinity, etc. [2,3], Therefore, archaea are good model systems for studying life in typically stressful environments [2]. In addition to their unique characteristics, archaea present genomic organization very similar to bacteria, while the information processing system has similar features to the eukaryotic machinery [4,5,6]. In accordance with their often-unusual lifestyle, laboratory cultivation of archaeal organisms is laborious and has historically limited the investigation of molecular mechanisms operating in its members. For the same reason, the implementation of molecular tools for genetic manipulation of archaea has been limited, but new alternatives have been generated with the advent of the genomic era [2,7]. Due to the genomic organization similar to bacteria, existing genetic tools for archaea are plasmid-based vectors harboring replication origins from both the archaeal organisms and *E*. *coli*, the later allowing the genetic manipulation of the vector using recombinant DNA technology [2,5,7].

With the widespread use of high-throughput technologies experienced in the last years, different archaea have been targeted in systems biology studies. In this sense, the halophilic archaeon *Halobacterium salinarum* has been intensively studied using several omics approaches [8, 9 ,^10^,^11^,^12^,^13^] and the deep level of information gathered led to the formulation of an accurate predictive model of its gene regulatory network [14,15]. This predictive model demonstrates the potential of integrating high-throughput data to get multi-level understanding of the molecular mechanisms operating in living organisms. While significant progresses in the post-genomic era have been experienced, a still existing limitation related to *in vivo* functional studies is the absence of optimized, high performance genetic tools for the manipulation of the target organism. This fact is not restricted to archaea, and several attempts have been made to overcome this limitation by generating new standardized genetic tools for diverse model organisms, from bacteria to eukaryotes [16,17,^18^,^19^]. The underlying principle is that the genetic tools should be minimal (i.e. devoid of unnecessary sequences), modular and sequence optimized to remove deleterious features (such as useful restriction sites from functional parts, which should be exclusive to the multiple cloning site-MCS). These principles have emerged mainly in the field of synthetic biology, where sequence optimization is a critical step to accomplish the assembling of large regulatory circuits [20]. In this work, we present the construction and characterization of a set of modular and optimized vectors to genetic engineer the halophilic archaeon *H*. *salinarum*. The innovation of the tools presented here is that different functionalities (e.g. cloning system, suicide vector, expression system and promoter probing) are placed under a standard format that allows interchangeability of the components and future vectors development, with the incorporation of new functional modules. We believe that our modular system will consistently speed up *in vivo* functional studies in this archaeon.

## Results and Discussion

### Designing a modular vector architecture for Archaea

**Fig 1:**
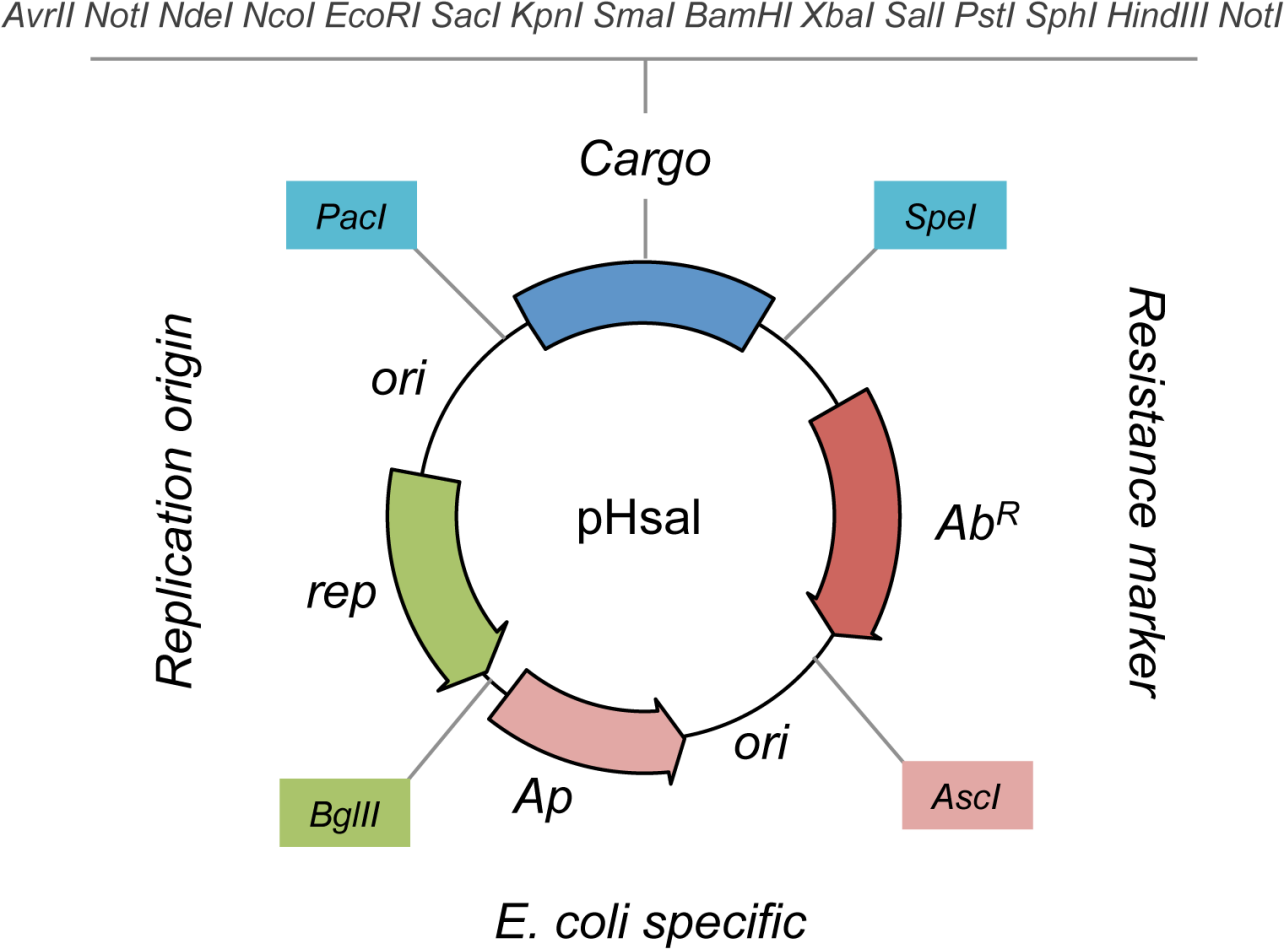
Modular design of pHsal vector series. The format is inspired in the SEVA platform [18] and is divided into four modules: (i) the *E*. *coli* specific part (origin of replication and ampicillin resistance gene); (ii) the replication origin; (iii) the resistance marker (tagged as Ab^R^) and (iv) the cargo. Each module is flanked by a unique restriction site that allows the easy replacement of a segment by a new sequence (for example, different resistance markers or origin of replications). The cargo is the main region of the vectors since it is used for cloning the fragments of interest.

Standard formats for genetic tools have been proposed for different organisms to facilitate the implementation of synthetic circuits and the distribution of material between different laboratories [18,21,22,23]. As a starting point for our modular archaeal vectors, we inspired our design in the SEVA format (Standard European Vector Architecture), which was used to construct modular vectors for a broad range of gram-negative bacteria [18]. The design used here is represented in Fig 1. As shown in the schema, the pHsal vectors have four modules, each one with specific functionalities. The first module allows the replication of the vectors in the *E*. *coli* host to facilitate genetic manipulation of the plasmids: it is formed by the multi-copy ColE1 replication origin and the *bla* gene for ampicillin resistance. Both segments are taken from pUC19 vector [24]. The second element is a replication origin that confers autonomous maintenance in the archaeal host. We used the minimal origin from pGRB1 plasmid [25], which is widely used in existing vectors for *H*. *salinarum* [2]. The third element is a resistance marker that allows the selection of *H*. *salinarum* strains harboring the plasmids. We used the mevinolin resistance marker *mev* ^*R*^, which has been successfully used in *H*. *salinarum* [2,7]. Finally, the fourth module is the Cargo, which represents an extensive MCS flanked by universal M13 primers. This architecture facilitates cloning procedures and further confirmation steps, such as digestions and sequencing. As shown in Fig 1, each functional module is flanked by a unique restriction site that can be used to replace these elements by alternative variants [18]: the cargo module is flanked by *Pac*I and *Spe*I restriction sites, the resistance marker by *Spe*I and *Asc*I, the archaeal replication origin by *Pac*I and *Bgl*II and the *E*. *coli*’s specific part by *Bgl*II and *Asc*I.

**Fig 2:**
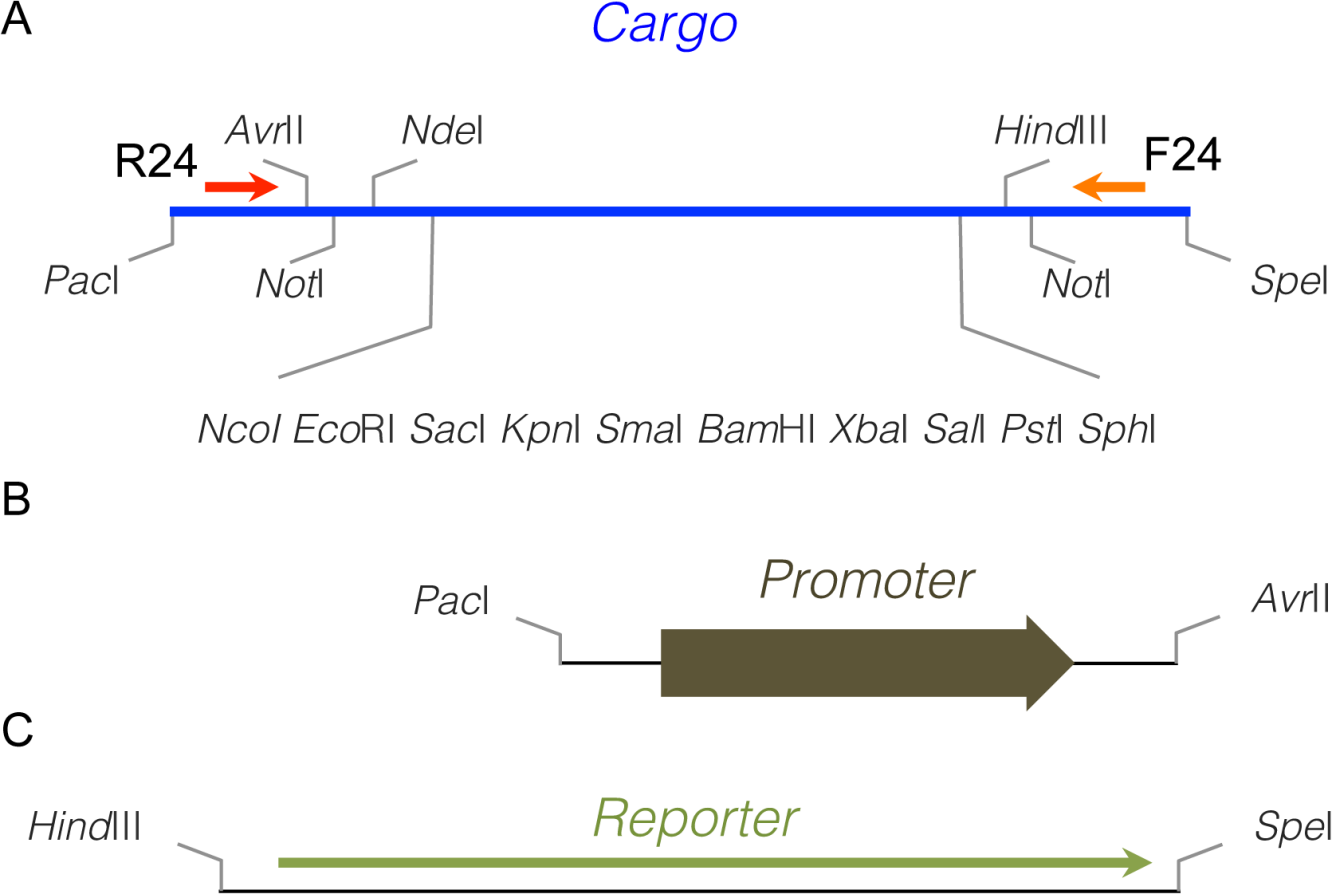
Schematic representation of the cargo architecture. (**A)** The basic cargo is a 150 nt long sequence containing an extensive multiple cloning site and a pair of universal primers (F24 and R24), allowing the user to easily clone and check the sequence of interest. (**B)** The expression systems of the pHsal series are cloned as *Pac*I/*Avr*II fragments at the 5’-end of the MCS. (**C)** The reporter systems for promoter probing are cloned as *Hind*III/*Spe*I fragments at the 3’-end of the MCS. With this design, the fragments of interest could be cloned using any of the restriction sites from *Avr*II to *Hind*III, always considering the directionality of the expression and reporter systems.

Since unique restriction sites should be exclusively used to replace the functional modules, any fragment used to construct the vectors should be first sequence-edited to eliminate these sites, as well as those restricted to the MCS region. The modular design used in pHsal series also includes a specific architecture for other functional elements such as universal primers and insertion of expression and reporter systems (Fig 2). In this case, universal primers (F24 and R24) are placed between the flanking restriction sites (*Pac*I or *Spe*I) and the nearest restriction sites in the MCS (Fig 2A). Similarly, any expression element (either a promoter or a promoter plus its regulator) can be inserted between *Pac*I and *Avr*II sites in the MCS (Fig 2B), while reporter systems are introduced at the end of this region between *Hind*III and *Spe*I restriction sites (Fig 2C). The next sections describe the construction of functional modules and the implementation of new functional elements such as promoters and reporter systems for *H*. *salinarum*.

### Construction of a set of modular vectors for *H*. *salinarum*

**Fig 3:**
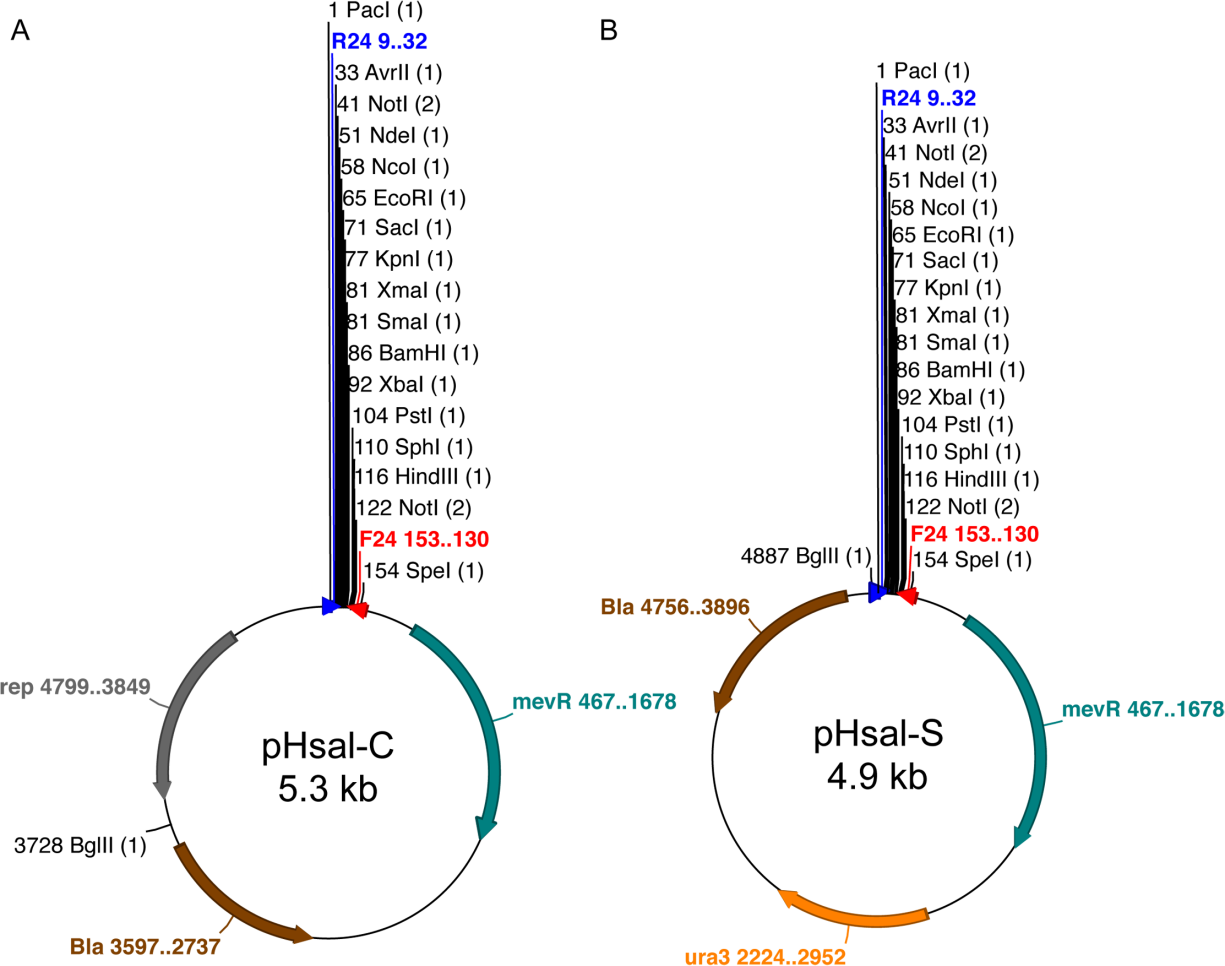
Physical maps of the modular cloning (pHsal-C) and suicide (pHsal-S) vectors. The main features of the vectors are represented, along with their relative positions. (A) pHsal-C is formed by the cargo, a *mev* ^*R*^ resistance marker and an origin for autonomous replication in *H*. *salinarum*, while (B) pHsal-S is endowed with a mev^R^ and an *ura3* marker and is devoid of replication origins for this archaeon. Yet, both vectors have the fragment with the Ap^R^ resistance marker (*bla* gene) and the ColE1 replication origin for replication and selection in *E*. *coli* host.

Based on the modular design presented above, we constructed a series of minimal, modular vectors for *H*. *salinarum*. The maps of the two main vectors are presented in Fig 3. In the pHsal series, pHsal-C is a 5.3 kb cloning vector that has a mevinolin resistance marker and a pGRB1 replication origin for autonomous maintenance in *H*. *salinarum* (Fig 3A). This vector is devoid of any expression system. and can be used with user-defined promoters and genes. Two variants of pHsal-C were constructed for different applications: pHsal-E and pHsal-GFP. The vector pHsal-E harbors an expression system based on a variant of the strong *fer2* promoter (see below) that is cloned between the *Pac*I and *Avr*II sites in the MCS (S1A Fig at the Supporting Information file 1). This vector allows high-level, constitutive expression of heterologous genes in *H*. *salinarum*. The pHsal-GFP vector is a variant of pHsal-C that harbors a GFP reporter gene between *Hind*III and *Spe*I sites and can be used to quantify promoter activities in *H*. *salinarum* (S1B Fig).

While the three vectors described above allow autonomous maintenance of the genes of interest in *H*. *salinarum*, some applications require the modification of chromosomal sequences to generate stable and permanent genotypes. Construction of knockouts strains, tagging of proteins with an epitope or replacement of a wild type gene by a mutant variant [2,7,13] require the use of a suicide vector with a marker for counter selection, such as the *ura*/*pyr* strategy [26]. We have thus constructed a vector named pHsal-S, which is devoid of a replication origin for *H*. *salinarum* and has both the *mev* ^*R*^ resistance marker for positive selection and a sequence-optimized *ura3* (VNG1673G) gene for counter selection with 5-FOA [26]. It is important to highlight that the pHsal-S vector not only has a modular architecture and an extended MCS, but is also about 1.6 kb shorter than the currently available vector for knockout generation and protein tagging [13]. This size reduction is possible since only minimal functional sequences were used, facilitating the manipulation of the vector *in vitro*. All these new modular vectors, along with their functional components were tested *in vivo* in *H*. *salinarum* and were found to be functional in this organism, as described in the next sections. Taken together, these new vectors provide a set of valuable genetic tools for engineering *H*. *salinarum* that could significantly aid functional studies in this model organism.

### Sequence optimization of a strong expression system for *H*. *salinarum*

**Fig 4:**
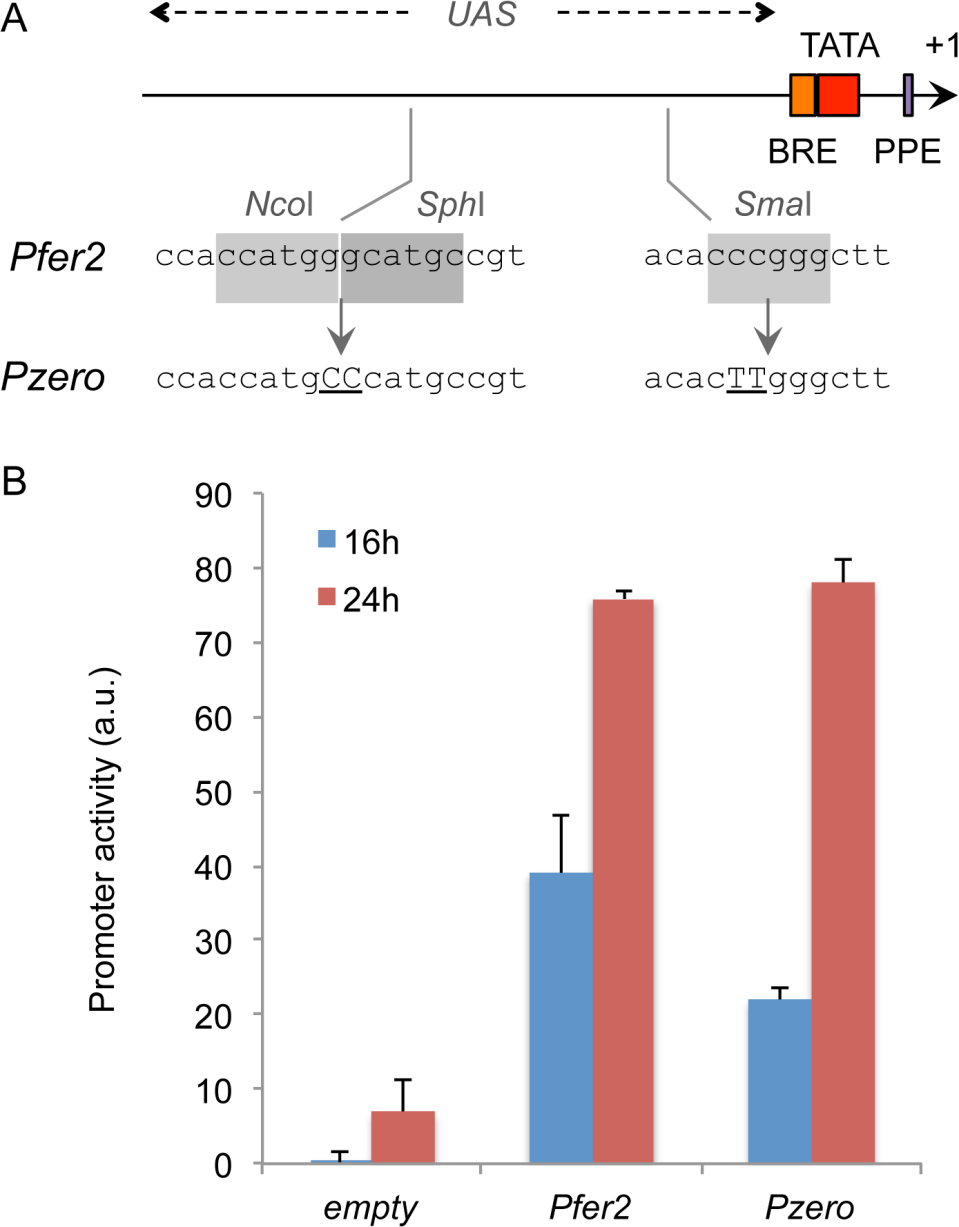
Sequence optimization of a strong promoter sequence for *H*. *salinarum*. **(A)** A 200 bp long sequence for the *fer2* promoter was sequence edited by overlapping PCR to eliminate 3 restriction sites for the enzymes *Nco*I, *Sph*I and *Sma*I. The resulting edited sequence was named *Pzero*, cloned in front of a promoterless GFP reporter gene and inserted in *H*. *salinarum* NRC-1. In the schema, the TATA box, BRE, PPE and UAS elements are represented [28]. (**B)** For the analysis of promoter activity, *H*. *salinarum* strains were assayed at mid (16h) and late (24h) exponential phases and the activity of the edited promoter was compared to the wild type sequence (*Pfer2*). An empty vector with no promoter cloned was used as control to check basal GFP expression of the system. Vertical bars represent standard deviation from experiments performed in triplicate.

As mentioned above, pHsal-E vector allows the expression of heterologous fragments in *H*. *salinarum* from a strong, constitutive *fer2* promoter [27] that was sequence edited to eliminate useful restriction sites. Fig 4A shows a schematic representation of the *fer2* promoter region (Pfer2), where TATA box, BRE and PPE regions [28] are represented. In the Pfer2 wild-type sequence, the region 150 bp upstream of BRE encompasses the *Upstream Activation Region* (UAS), where we identified three restriction sites for the enzymes *Nco*I, *Sph*I and *SmaI*. We used mutagenic PCR to change four bases in the wild type sequence, eliminating these restriction sites. The mutated UAS sequence was assembled by PCR and cloned into a GFP reporter vector, generating the promoter *Pzero* (Fig 4A). To check the functionality of the sequence-edited promoter, we introduced reporter plasmids with the wild type *Pfer2* or with *Pzero* into *H*. *salinarum*. As a control, a plasmid devoid of any promoter was also introduced to determine the basal activity of the system. Recombinant *H*. *salinarum* strains harboring the plasmids of interest were grown in CM media and the promoter activities were assayed at mid (16h) and late (24h) exponential phase. As shown in Fig 4B, the sequence-optimized *Pzero* displayed a promoter activity very similar to the wild type *Pfer2*, showing that the mutations introduced into the promoter did not affect significantly its activity. Thus, the data provided here show the construction of sequence optimized, strong expression system for high-level expression of heterologous genes in *H*. *salinarum*.

### Validation of the suicide vector for chromosomal tagging

**Fig 5:**
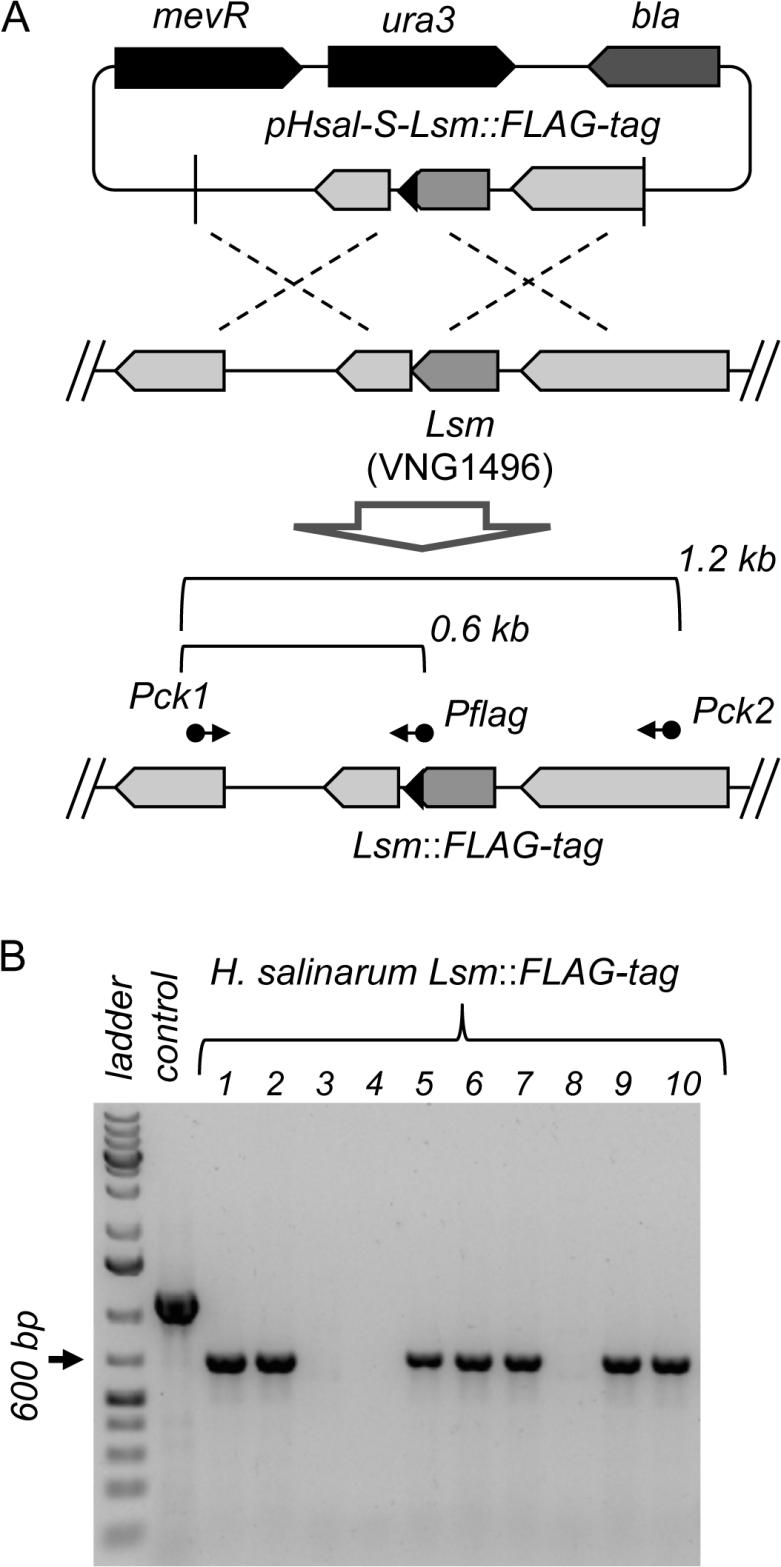
Construction of *H*. *salinarum* strains with chromosomal tags. **(A)** A suicide plasmid pHsal-S-Lsm::FLAG-tag, harboring a modified flanking region of the Lsm gene to introduce a FLAG-tag epitope, was transformed into *H*. *salinarum*. Double recombination events were selected and correct incorporation of the FLAG-tag was checked using primers *Pck1* and *Pflag*. As a control, primers *Pck1/Pck2* were used to amplify the whole flanking region of the Lsm coding gene. (**B)** PCR validation of the FLAG-tag incorporation. Ten (numbered from 1 to 10) independent colonies were selected and screened using primers *Pck1/Pflag*, which should give rise to an amplification band of ~ 600 bp. The 1.0 kb DNA ladder is shown on line 1, while line 2 shows the amplification control, representing the flanking region obtained using primers *Pck1/Pck2*.

To demonstrate the efficacy of the new tools constructed here for genome editing, we used the pHsal-S vector for the generation of *H*. *salinarum* strains harboring chromosomal tags in genes of interest. As a proof of concept, we targeted the RNA chaperone Lsm encoded by the gene VNG1496G. We selected the FLAG-tag since it is a widely used and short (8 aa) tag peptide [29], with reduced chances of affecting the structure of the target protein. For the construction of the tagged strain, flanking regions of 500 bp upstream and downstream of the stop codon of the VNG1496G gene were PCR amplified and assembled into a 1.0 kb fragment by recombinant PCR. In this procedure, the homology primers of the recombinant PCR harbor the nucleotide sequence coding the FLAG-tag, allowing the insertion of the tag at the 3’-end of the gene. The final recombinant fragment was then cloned into pHsal-S vector and the resulting plasmid was used to transform *H*. *salinarum*. Recombinant strains with the plasmid integrated into the chromosome were selected by plating the transformation in selective media supplemented with mevinolin. After the appearance of colonies in the selective media, a single one was picked and inoculated into liquid media without selective pressure to allow the second event of recombination. After saturation of the liquid culture, cells were plated in solid media with 5-FOA to counter-select non-recombinant strains [13]. Colonies able to grow under these conditions were checked by PCR to verify if the tag was correctly inserted into the chromosome or if the strains were able to revert to the wild-type genotype (Fig 5). As shown in Fig 5B, we detected 7 out of 10 colonies that were positive for the correct incorporation of the FLAG-tag. This result highlights the applicability of the new suicide vector pHsal-S for the generation of stable genotypes in *H*. *salinarum*.

## Conclusions

The use of new technologies to get large-scale access on molecular mechanisms operating in living organisms has changed our way to see biological systems. However, the progress to get experimental data in the field of systems biology is not always followed by the development of experimental tools to allow large-scale engineering of the target organism and test the hypotheses raised by systems approaches. By the same token, synthetic biology applications also require the development of high-performance tools to allow large-scale assembly and implementation of synthetic circuits [30]. Standardized genetic tools have already been developed for bacteria [18,21] and eukaryotes [19]. As far as we are concerned, the modular vectors presented here are the first attempt to build modular genetic tools for archaea. We targeted the halophilic archaeon *H*. *salinarum*, a model organism in systems biology and we anticipate that many of the hypotheses generated so far will now be more easily addressed using these tools. Furthermore, we encourage other researches working in archaea to adopt similar strategies to construct modular vectors for different organisms, perhaps using the pHsal vectors as a starting point. Finally, we stress that the vectors developed here are freely available.

## Materials and Methods

### Strains, plasmids and growth conditions

*Halobacterium salinarum* NRC-1 cells were grown in enriched complex media (CM) consisting of 25% NaCl, 2% MgSO_4_.7H_2_O, 0.2% KCl, 0.3% Na-citrate and 1% peptone, at 37°C under light and constant agitation of 225 rpm [31]. When required, the media was supplemented with 20 μg/mL of mevinolin (A.G. Scientific, San Diego, CA) or 5-fluoroorotic acid (5-FOA, 300 μg/mL). Cultivation and transformation of *H*. *salinarum* were performed according to standard protocols [31]. *Escherichia coli* DH5α cells were grown at 37°C with air shaking at 225 rpm in Luria-Bertani media (LB; 1% triptone, 0.5% yeast extract, 0.5% Na-chloride). When required, the media was supplemented with 100 μg/mL of carbenicilin (Sigma) to ensure plasmid retention. Synthetic DNA sequences were obtained from GeneArt (Life Technologies). All DNA manipulation techniques were performed according to standard protocols.

### General cloning procedures

For the construction of the pHsal vector series, some initial vectors were constructed as starting points for assembling the modular vectors (**Table 1**). First, a ~1.8 kb fragment containing the *bla* gene and the ori ColE1 was PCR amplified using primers 5-pUC-BglII and 5-pUC-AscI that incorporate *Bgl*II and *Asc*I restriction sites in the flanking regions. This fragment was digested and ligated to a 150 bp synthetic DNA fragment (GeneArt) containing the MCS of the system, generating the pRzero vector. Next, the pRzero vector was digested with the enzymes *Asc*I and *Spe*I and ligated to a 1.2 kb long synthetic sequence of the optimized *ura3* gene (named *ura3-2*.*0*, *GeneArt*) digested with the same enzymes, generating the vector pRU. In turn, the *mev* ^*R*^ gene was PCR amplified from the plasmid pMTF1025GFP_CHA with primers 5-mev-SpeI and 3-mev-XbaI, digested with the enzymes *Spe*I and *Xba*I and cloned into a pRU vector previously digested with *Spe*I. From the resulting colonies, a recombinant vector that regenerated the single *Spe*I site at the 5’-end of the *mev* ^*R*^ gene was named pHsal-S (Fig 3B), which is a suicide vector for the insertion of chromosomal modifications in *H*. *salinarum*. For the generation of the pHsal-S-Lsm::FLAG-tag vector, a 1.0 kb fragment containing the flanking regions of the Lsm gene (VNG1496G) was assembled by recombinant PCR using an upstream fragment amplified using primers 5-Lsm-EcoRI/3-flag and a downstream fragment, obtained with primers 5-flag/Pck2. The two fragments were joined using primers 5-Lsm-EcoRI /Pck2 and cloned into pHsal-S vector in *Eco*RI and *Hind*III restriction sites. The resulting recombinant vector was used to transform *H*. *salinarum* and generate the tagged version of Lsm as described previously [13].

**Table 1:** Strains, plasmids and primers used in this study.

Strains	Description	Reference
*E*. *coli* DH5α	*F* ^*-*^ *endA1 glnV44 thi-1 recA1 relA1 gyrA96 deoR nupG Φ80dlacZΔM15 Δ(lacZYA-argF)U169*, *hsdR17(r*_*K*_^*-*^ *m*_*K*_^*+*^ *)*, *λ* ^*–*^	[32]
*H*. *salinarum* NRC-1	Wild type reference strain of *Halobacterium salinarum*	[31]
Plasmids		
pMTF1025GFP_CHA	*Ap* ^*R*^, *ori ColE1*, *Mev* ^*R*^, *ori pGRB1*; *E*. *coli*-*H*. *salinarum* shuttle vector with *Pfer2* and GFP reporter gene	Baliga’s lab
pUC19	*Ap* ^*R*^, *ori ColE1*, lacZα; standard cloning vector	[24]
pRzero	*Ap* ^*R*^, *ori ColE1*; a 2.0 kb, minimal *E*. *coli*’s vector with synthetic MCS	This study
pRU	*Ap* ^*R*^, *ori ColE1*, *ura3-2*.*0*; a variant of pRzero containing an optimized *ura3* gene	This study
pRO	*Ap* ^*R*^, *ori ColE1*, *ori pGRB1*; a variant of pRzero containing a minimal pGRB1 ori for autonomous replication in *H*. *salinarum*	This study
pHsal-S	*Ap* ^*R*^, *ori ColE1*, *ura3-2*.*0*, *Mev* ^*R*^; a variant of pRU with a 1.8 kb fragment containing wild type *Mev* ^*R*^ gene. Suicide vector in *H*. *salinarum*	This study
pHsal-C	*Ap* ^*R*^, *ori ColE1*, *Mev* ^*R*^; a variant of pRO with a 1.8 kb fragment containing sequence optimized *Mev* ^*R*^ gene. Modular shuttle vector for *H*. *salinarum*	This study
pHsal-*E*	*Ap* ^*R*^, *ori ColE1*, *Mev* ^*R*^; a variant of pHsal-C with *Pzero* promoter cloned as a *Pac*I/*Avr*II fragment. Expression vector	This study
pHsal-*GFP*	*Ap* ^*R*^, *ori ColE1*, *Mev* ^*R*^; a variant of pHsal-C with hGFP gene cloned as a *Hind*III/*Spe*I fragment. Promoter probe vector	This study
Primers	**Sequence (5’-3’)**	**Reference**
5-pUC-BglII	GGCGCAGATCTAGGTGGCACTTTTCGGG	This study
3-pUC-AscI	CGCCGGCCGGCCGAGCAAAAGGCCAGCAAAAGGC	This study
5-mev-SpeI	GCGGACTAGTGACCCGCGTCTCGACG	This study
3-mut1	GACGAGCAGTCGGCGGGCCTCGGCGGCGGTG	This study
5-mut1	CACCGCCGCCGAGGCCCGCCGACTGCTCGTC	This study
3-mut2	GACGGAGCCGCCGTCCACGCTGACGGGGCCG	This study
5-mut2	CGGCCCCGTCAGCGTGGACGGCGGCTCCGTC	This study
3-mev-AscI	CCGCGGCGCGCCGCGTGTGAAGAGTGGCATGG	This study
3-mev-XbaI	CCGCTCTAGAGCGTGTGAAGAGTGGCATG	This study
5-Pfer2-KpnI	GCGCGGTACCGGCCGGCAGCACCTG	This study
5-Pfer2-M	CATACCCCCACCATGCCCATGCCGTGGATAA	This study
3-Pfer2-M	TTATCCACGGCATGGGCATGGTGGGGGTATG	This study
3-Pfer2	TTGTGTGACCGCCATCACG	This study
5-GRB1-PacI	GCGGTTAATTAAGGACCAATACTGGCTCCACGC	This study
3-GRB1-BglII	CCGCAGATCTACTCATCGACATCCCAATCTGC	This study
5-Pzero-PacI	GCGCTTAATTAAGGCCGGCAGCACCTG	This study
3-Pzero-AvrII	GCGCCCTAGGGGTATGTGCAGAGTTCGGCTTCC	This study
5-GFP-HindIII	GCGCAAGCTTATGAGTAAAGGAGAAG	This study
3-GFP-SpeI	GCGCACTAGTTTAGTCGTCTAAAGCG	This study
5-FLAG	GACTACAAAGACGATGACGACAAG	This study
3-FLAG	CTTGTCGTCATCGTCTTTGTAGTC	This study
5-Lsm-EcoRI	ATCTGAATTCGGCGACGGCCCCGTGGTGAT	This study
3-Eflag	TTACTTGTCGTCATCGTCTTTGTAGTCTGGTTTGATGGTGACG	This study
5-Eflag	GACGATGACGACAAGTAACTGGCGCAGGAACCCCGA	This study
Pck2	AGATAAGCTTTGGGCGACGATGCCCGCCGA	This study
Pck1	TTGGGTTCGACCTCGACGTG	This study
Pflag	CTTGTCGTCATCGTCTTTGTAGTC	This study

For the construction of modular vectors able to replicate autonomously in *H*. *salinarum*, a ~1.6 kb fragment containing the pGRB1 replication origin [25] was PCR amplified from the pMTF1025GFP_CHA vector and cloned as a *Bgl*II/*Pac*I fragment into the pRzero vector, previously digested with the same enzymes. The resulting plasmid was named pRO and used in the next steps. In order to create a sequence-edited version of the *mev* ^*R*^ gene, three DNA fragments were PCR amplified using pMTF1025GFP_CHA vector as template with primers 5-mev-SpeI/3-mut1 (fragment 1), 5-mut1/3-mut2 (fragment 2) and 5-mut2/3-mev-AscI (fragment 3). These primers allow the elimination of an *Asc*I and a *Sal*I restriction sites present in the wild type sequence of the gene. Next, the three fragments were used in a PCR reaction with primers 5-mev-SpeI/3-mev-AscI to reconstruct the complete *mev* ^*R*^ gene by overlapping PCR. The full 1.8 kb fragment was agarose gel purified and cloned using *Asc*I and *Spe*I enzymes into the pRO vector, generating pHsal-C (Fig 3A). This vector is able to replicate autonomously in *H*. *salinarum* under the selection for mevinolin resistance.

For the construction of pHsal-E, we first generated and validated a sequence-edited version of the *fer2* promoter of *H*. *salinarum*. For this, two fragments of the *Pfer2* sequence were PCR amplified using primers 5-Pfer2-KpnI/3-Pfer2-M and 5-Pfer2-M/3-Pfer2, and assembled into a single fragment in a second round of PCR reaction with primers 5-Pfer2-KpnI/3-Pfer2. This ~100 bp fragment was digested with KpnI and cloned into pMTF1025GFP_CHA vector previously digested with KpnI/SmaI enzymes, generating vector pMTF1025GFP-*Pzero*. Once *Pzero* activity was validated *in vivo*, the synthetic promoter was PCR amplified using primers 5-Pzero-PacI/3-Pzero-AvrII and cloned as a *Pac*I/*Avr*II fragment into pHsal-C, generating pHsal-E, which is an expression plasmid based on the strong *Pzero* promoter activity. Finally, for the construction of a GFP-based vector for promoter probing, a 0.8 kb fragment containing the GFP sequence was PCR amplified using pMTF1025GFP_CHA as template and primers 5-GFP-HindIII/3-GFP-SpeI. This fragment was cloned with enzymes *Hind*III/*Spe*I into pHsal-C, generating pHsal-GFP. All vector sequences are available at our website (http://labisismi.fmrp.usp.br/index.php/en/resen) and as S1 File. All plasmids are available upon request by e-mail.

### GFP reporter assays

To quantify promoter activity, single colonies of *H*. *salinarum* strains with different reporter plasmids were inoculated in 5 mL of liquid CM supplemented with 20 μg/mL mevinolin and incubated for 5 days. After pre-growth, cells were diluted to an OD_600_ ~0.05 in fresh media and grown in standard conditions. At two specific time points (16 and 24 hours), samples (1.8 mL) were taken and the OD_600_ measured. Sample cells were centrifuged for 5 min at 13,000 rpm and the supernatant was discarded. Cells were then resuspended in 1.8 mL of GFP assay buffer (10mM Tris-HCl, pH 7.5) and mixed vigorously to allow cell lysis. After lysis, cell debris were removed by centrifugation and samples were analyzed in a RF-5301PC fluorimeter (Shimadzu). GFP assays were performed using wavelengths of 488 nm for excitation and 510 nm for emission. Promoter activities were calculated by normalizing the measured fluorescence by the initial cell density (fluorescence/OD_600_). A control strain of *H*. *salinarum* without the reporter plasmid was used to calculate the auto-fluorescence of the cells, and these background values were subtracted from the promoter activities.

## Supporting Information

S1 Fig Physical maps of the modular expression (pHsal-E) and reporter (pHsal-GFP) vectors. (PDF)

S1 File pHsal vector sequences. (TXT)
