# Supplementary material for: Development of New Modular Genetic Tools for Engineering the Halophilic Archaeon Halobacterium salinarum
Source: PLoS One. 2015 Jun 10;10(6):e0129215. doi: 10.1371/journal.pone.0129215 (PMC4465625; doi:10.1371/journal.pone.0129215)
Supplement: S1 Fig — (PDF) [file pone.0129215.s001.pdf]

Development of new modular genetic tools for engineering the halophilic archaeon *Halobacterium salinarum*

Rafael Silva-Rocha, Marjorie C. Pontelli, Gilvan P. Furtado, Livia S. Zaramela and Tie Koide*

Supplementary Material

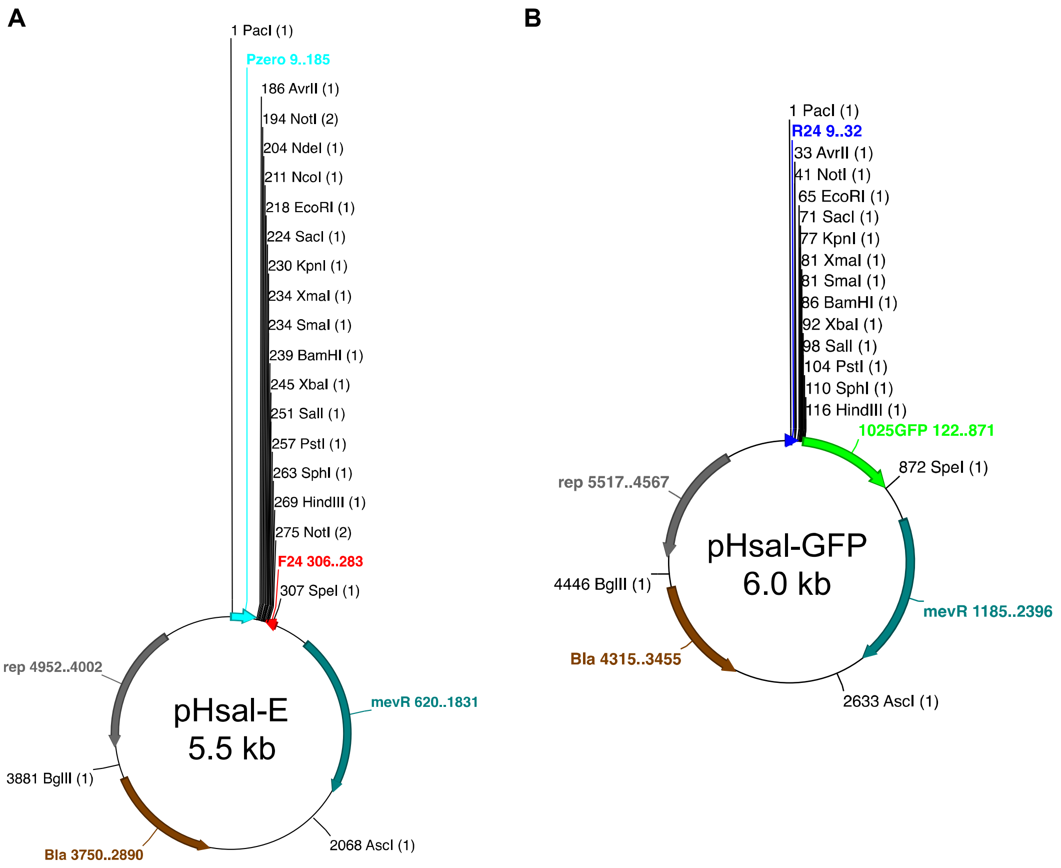

Figure S1. Physical maps of the modular expression (pHsal-E) and reporter (pHsal-GFP) vectors. The main features of the vectors are represented, along with their relative positions. Both pHsal-E and pHsal-GFP are formed by the cargo, a mev^R resistance marker and an origin for autonomous replication in *H. salinarum* and a fragment with the Ap^R resistance marker (*bla* gene) and the ColE1 replication origin for replication and selection in *E. coli* hosts. **A)** pHsal-E, which has a strong expression system based on the *Pzero* promoter. **B)** pHsal-GFP, which has a GFP reporter gene for promoter probing in *H. salinarum*.
